# High Morbidity and Mortality Among Patients With Sentinel Admission for Injection Drug Use-Related Infective Endocarditis

**DOI:** 10.1093/ofid/ofz089

**Published:** 2019-03-01

**Authors:** P Alexander Leahey, Mary T LaSalvia, Elana S Rosenthal, Adolf W Karchmer, Christopher F Rowley

**Affiliations:** 1 Division of Infectious Diseases, Beth Israel Deaconess Medical Center, Harvard Medical School, Boston, Massachusetts; 2 Department of Infectious Diseases, Kaiser Permanente Northwest, Clackamas, Oregon; 3 Division of Clinical Care and Research, Institute of Human Virology, University of Maryland School of Medicine, Baltimore, Maryland

**Keywords:** EuroSCORE, infective endocarditis, injection drug use

## Abstract

**Background:**

Hospitalizations for individuals with injection drug use-related infective endocarditis (IDU-IE) represent an increasing portion of all patients with endocarditis. This study describes the evolving trends in demographics, clinical characteristics, rates of surgical intervention, and mortality among patients hospitalized with IE, comparing those with and without injection drug use.

**Methods:**

This is a retrospective cohort study of patients admitted between January 1, 2007 to June 30, 2015 at a tertiary care center in Boston, Massachusetts. Endocarditis was defined by *International Classification of Diseases, Ninth Revision* code and verified by the modified Duke Criteria for IE. The clinical characteristics, microbiology, site of infection, complications of IE, and outcome were all abstracted by chart review. Rates of surgical consultation and surgical intervention within 90 days of admission were obtained, and assessment of surgical risk calculated was by EuroSCORE II (euroscore.org/calc). Subsequent hospitalizations for all causes were also reviewed.

**Results:**

Injection drug use-related infective endocarditis occurred in younger patients with lower rates of diabetes, renal dysfunction, and prior cardiothoracic (CT) surgery than those without IDU. Injection drug use-related infective endocarditis was associated with higher rates of complications, CT surgery consultation, and surgery within 90 days for absolute surgical indication. Readmissions for endocarditis occurred in 20% of IDU-IE patients and 9% of those with non-IDU IE. All-cause 1-year mortality rates were similar (IDU-IE 16%, non-IDU IE 13%; *P* = .58).

**Conclusions:**

Despite younger age, fewer medical comorbidities, and fewer prior cardiac surgeries, all-cause 1-year mortality was similar for patients after sentinel admission for IDU-IE compared with non-IDU IE. Interventions in the acute hospital setting and after discharge are needed to support patients with IDU-IE, focusing on harm reduction and treatment of addiction to reduce the unexpectedly high mortality of this young population.

Injection drug use (IDU) is responsible for an increasing number of cases of infective endocarditis (IE) in the United States, an observation that correlates with the increase in overdoses and overdose deaths secondary to opioid and heroin use [1, 2]. Injection drug use is a well established risk factor for serious infections such as IE [3–5], and persons with IDU-associated IE (IDU-IE) who require cardiac surgery have previously been reported to suffer worse outcomes, including higher rates of reinfection, reoperation, and mortality [6–8] than those individuals with endocarditis without IDU (non-IDU IE).

The reason for the reported difference in clinical outcomes in individuals with IDU-IE is unclear, particularly because these patients typically are younger with fewer comorbidities. It is possible that patients who present with IDU-IE face a more challenging clinical course due to the virulence of the responsible pathogen, embolic phenomena, coinfections, continued drug use, or different care received with respect to timing of surgical intervention when warranted [9]. Our study aims to evaluate the trends in demographics, microbiology, surgical intervention, and outcomes among patients hospitalized with IDU-IE compared with those with non-IDU-IE at our urban tertiary care center. This will assist us in identifying the current state of treatment for these individuals as well as to optimize care for this patient population at high risk of poor outcome.

## METHODS

### Study Design and Patient Population

We performed a retrospective review of individuals admitted from January 1, 2007 to June 30, 2015 with a diagnosis of IE at a large tertiary care center in Boston, Massachusetts. These dates were selected based on availability of a complete electronic medical record. The Committee on Clinical Investigations at the Beth Israel Deaconess Medical Center approved the study.

### Data Collection

Individuals with a primary diagnosis of IE at the time of hospital discharge for sentinel IE admission were identified by searching for *International Classification of Diseases, Ninth Revision* codes (112.81, 421.0, 421.1, 421.9, 424.9, 424.91, 424.99). Charts were reviewed to confirm the presence of definite IE as defined by modified Duke criteria [10]. Records were then reviewed to ascertain active IDU within 3 months of the sentinel IE admission. In these patients, IDU was considered the predisposition for IE and thus defined IDU-IE.

Medical records were additionally reviewed for demographic and baseline clinical information, microbiology, site of infection, complications of IE, and death. Microbiology was determined by detection of organism in blood cultures, culture of vegetation/valve specimen, or universal polymerase chain reaction of cardiac valve tissue. Left-sided infection was defined as echocardiographic evidence of IE exclusively involving the aortic or mitral valves and associated left atrium/ventricle structures, whereas right-sided infection was defined as IE exclusively involving the pulmonary or tricuspid valves and associated right atrium/ventricle structures. Complications of IE were categorized according to hemodynamic compromise (pulmonary edema, pressor requirement, left ventricular ejection fraction ≤30%, mitral valve preclosure), paravalvular abscess (detected by echocardiography or during surgical intervention), failure of antibiotics (7 or more days of positive blood cultures despite appropriate antimicrobial therapy), prosthetic valve dysfunction (valve hypermobility, unseated valve), extracardiac emboli (pulmonary and systemic), unresponsive organism (ie, resistant Gram-negative rod, *Candida* species), and vegetation greater than 1 cm in diameter as detected by echocardiography. Death was determined by evaluation of the electronic medical record, public obituary records, and the Social Security Death Index ([SSDI] 1935-2014 [http://search.ancestry.com/search/db.aspx?dbid=3693).

### Surgical Intervention

Information was collected regarding cardiothoracic (CT) surgical consultation and CT surgery intervention within 90 days of sentinel IE admission. Absolute surgical indications were defined by the presence of hemodynamic compromise, paravalvular abscess, failed antibiotics, or prosthetic valve dysfunction.

Surgical intervention was defined as thoracotomy with valve repair, valve replacement, aortic root replacement, or Bentall procedure. Surgical risk factors at the time of surgical consultation included age, the presence of diabetes mellitus (insulin-dependent), renal dysfunction, prior cardiac surgery with thoracotomy, prior IE, or prosthetic cardiac valve. Additional surgical risk factors associated with mortality in adult cardiac surgery patients were collected to calculate a surgical risk score and determine quintiles of risk according to the EuroSCORE II multinational database (http://www.euroscore.org/calc.html) [11].

### Statistics

Descriptive statistics were performed with SAS, version 9.3 (SAS Institute, Cary, NC). Logistic regression to assess variables associated with surgical intervention was performed in all individuals with endocarditis who had an absolute indication for surgery. Variables used in the logistical regression for cardiac surgery performed within 90 days of sentinel IE admission included the following: gender, age, race, presence of paravalvular abscess, prosthetic valve, active IDU, extracardiac emboli, EuroSCORE (in quintiles), IE caused by *Staphylococcus aureus*, and IE involving only the right side of the heart. Univariate analysis was performed and all values with *P* ≤ .10 were included in the multivariate model.

## RESULTS

Between January 1, 2007 and September 30, 2015, 381 patients were admitted with definite IE, 103 (27%) of whom had IDU-IE. Baseline demographics and clinical characteristics are detailed in Table 1. The median age of patients with IDU-IE was 33 (interquartile range [IQR], 26–44) compared with 63 (IQR, 53–74) in non-IDU (*P* < .0001). There was a higher proportion of women among those with IDU-IE compared with non-IDU IE (46% vs 30%) with no difference in race, with white patients accounting for 76% of patients in the IDU-IE group. Hepatitis C virus infection was more common in patients with IDU-IE (66 [70%] vs 17 [16%]), although the prevalence of human immunodeficiency virus was similar (4 [4.8%] vs 7 [8.0%]). Patients with IDU-IE were less likely to have concurrent diabetes, renal dysfunction, and prior cardiac surgery.

**Table 1.** Demographics and Bacteriology of Patients With Infective Endocarditis With and Without Injection Drug Use

Characteristics	IDU-IE	Non-IDU IE	*P* Value
	n = 103	n = 278	
Age, years—median (IQR)	33 (26–44)	63 (53–74)	<.0001
Sex, n (%)			
Male	56 (54.4)	194 (69.8)	
Female	47 (46.6)	84 (30.2)	
Race, n (%)			
White	78 (76)	202 (73)	
Black	9 (9)	31 (11)	
Hispanic	6 (6)	19 (7)	
Asian	1 (1)	6 (2)	
Unknown	9 (9)	20 (7)	
HIV, n (%)	4/83 (5)	7/88 (8)	N/A^a^
HCV, n (%)	66/94 (70)	17/106 (16)	N/A^a^
DM, n (%)	3 (3)	47 (17)	.0001
Renal Function, n (%)			
Normal (GFR >85 mg/dL)	76 (74)	145 (52)	.0002
Moderately impaired (GFR 50–85 mg/dL)	12 (12)	47 (17)	
Severely impaired (GFR <50 mg/dL)	15 (15)	54 (19)	.001
Dialysis dependent	0 (0)	32 (12)	
Prior CT surgery n (%)	9 (9)	63 (23)	.003
Previous IE, n (%)	22 (21)	18 (7)	<.0001
Prosthetic valve, n (%)	9 (9)	45 (16)	.06
Microbiology, n (%)			
MSSA	48 (47)	61 (22)	<.0001
MRSA	19 (18)	36 (13)	.18
Streptococcus spp	17 (17)	89 (32)	.003
Enterococcus spp	7 (7)	38 (14)	.06
CoNS	1 (1)	17 (6)	.04
Gram negative	3 (3)	2 (1)	.09
Candida spp	4 (4)	6 (2)	.35
Polymicrobial/other	2 (2)	9 (3)	.50
Culture negative/unknown	2 (2)	17 (6)	.10

Abbreviations: CT, cardiothoracic; DM, diabetes mellitus; GFR, glomerular filtration rate; HCV, hepatitis C virus; HIV, human immunodeficiency virus; IDU IE, injection drug use-related infective endocarditis; IQR, interquartile range; MRSA, methicillin-resistant *Staphylococcus aureus*; MSSA, methicillin-sensitive *S aureus*; CoNS, coagulase-negative staphylococci.

^a^Denominator represents the number of tests performed. No statistical comparison was conducted due to large number of missing values in the non-IDU IE group.

The microbiologic cause for endocarditis is also illustrated in Table 1. *Staphylococcus aureus* was the most common species causing endocarditis in both groups; however, it was responsible for approximately twice the percentage of the cases in those with IDU (67 [65%] in IDU-IE and 97 [35%] in non-IDU IE), with methicillin-sensitive *S aureus* (MSSA) occurring more frequently than methicillin-resistant *S aureus* (MRSA) in both groups.

Table 2 presents the site of infection, IE complications, and comprehensive EuroSCORE. Injection drug use-related infective endocarditis versus non-IDU IE was more frequently right-sided (35 [35%] vs 15 [5%]) and more likely to involve both the left and right sides (10 [10%] vs 7 [3%]). Injection drug use-related infective endocarditis compared with non-IDU IE was more likely to be associated with a vegetation >1 cm (61 [59%] vs 84 [30%]; *P* < .01), systemic emboli (85 [83%] vs 135 [49%]; *P* = .04), and resistant organisms (8 [8%] vs 8 [3%]; *P* = .04). Cardiothoracic surgery was more frequently consulted in IDU-IE (74 [72%]) compared with non-IDU IE (155 [56%]). The presence of 1 or more absolute surgical indication was similar in both groups. Cardiothoracic surgical intervention within 90 days of sentinel admission was more frequent in cases of IDU-IE (40 [39%] vs 79 [28%]), which was statistically significant when surgical intervention was stratified by the presence of at least 1 absolute surgical indication (29 [64%] vs 43 [43%]; *P* = .02).

**Table 2.** Cardiac Valve Involvement, Cardiac Consultation, Surgery Performed, and Surgical Risk Stratification in Patients With Infective Endocarditis

Characteristics	IDU-IE	Non-IDU IE	*P* Value
Site of Infection	n = 103 (%)	n = 287 (%)	
No vegetation seen	12 (12)	16 (6)	.12
Left side	45 (44)	240 (86)	<.00001
Right side	36 (35)	15 (5)	<.00001
Both	10 (10)	7 (3)	.003
Surgical Indications			
None	3 (3)	59 (21)	<.0001
NYHA Class III of IV	27 (26)	76 (27)	.83
Hemodynamic compromise	39 (38)	83 (30)	.14
Paravalvular abscess	15 (15)	26 (9)	.19
Failed antibiotics	1 (1)	5 (2)	1
PV dysfunction	2 (2)	11 (4)	.52
Systemic emboli	85 (83)	135 (49)	<.0001
Unresponsive organisms	8 (8)	8 (3)	.04
Vegetation >1 cm	61 (60)	84 (30)	<.0001
CT surgery consultation	74 (72)	155 (56)	.005
Surgery performed	40 (39)	79 (28)	.06
Absolute surgical indication	45 (44)	101 (36)	.19
Absolute indication present and surgery performed	29 of 45 (64%)	43 of 101 (43%)	.02
Surgical Mortality Risk: EuroSCORE II Calculated for Those With Absolute Surgical Indication			
	n = 45 (%)	n = 101 (%)	
Low (0.5%–1.9%)	16 (36)	16 (16)	
Mild (2.0%–3.1%)	8 (18)	18 (18)	
Moderate (3.2%–5.5%)	7 (16)	19 (19)	
High (5.6%–10%)	7 (16)	16 (16)	
Very High (>10%)	7 (16)	32 (32)	

Abbreviations: CT, cardiothoracic; IDU IE, injection drug use-related infective endocarditis; NYHA, New York Heart Association; PV, pulmonary valve.

As detailed in Table 3, patients who were 60 years of age or less, those who had increased surgical mortality risk (increased EuroSCORE compared with lowest risk category except for high-risk group), large vegetations, and the presence of a paravalvular abscess had an increased odds of surgery within 90 days in the multivariate analysis. The presence of only right-sided IE was a negative predictor for surgery although it did not meet statistical significance (*P* = .06).

**Table 3.** Univariate and Multivariate Logistic Regression of the Odds of Cardiac Surgery Within 90 Days of the Sentinel Admission for Endocarditis for Individuals With an Absolute Surgical Indication

Characteristics	OR (95% CI)	*P* Value	aOR (95% CI)	*P* Value
Race (white vs other)	0.86 (0.42–1.76)	.69	—	—
Female	0.57 (0.29–1.15)	.12	—	—
Age (60 or less)	3.98 (1.93–8.21)	.0002	8.26 (3.20–21.36)	<.0001
*Staphylococcus aureus*	0.45 (0.23–0.90)	.02	0.52 (0.21–1.27)	.15
Active IDU	1.67 (0.82–3.38)	.16	—	—
Large vegetation	2.11 (1.08–4.11)	.03	2.88 (1.22–6.78)	.02
Paravalvular abscess	3.32 (1.57–7.05)	.002	2.91 (1.17–7.27)	.02
Euroscore Category				
Low risk	Reference	—	Reference	—
Mild risk	4.09 (1.34–12.49)	.02	3.87 (1.01–14.87)	.049
Moderate risk	2.20 (0.72–6.72)	.17	4.96 (1.12–20.70)	.03
High risk	1.31 (0.40–4.34)	.66	1.63 (0.38–6.99)	.51
Very high risk	3.50 (1.26–9.69)	.02	8.51 (2.13–33.94)	.002
Prosthetic valve dysfunction	1.18 (0.38–3.70)	.78	—	—
Systemic emboli	0.90 (0.46–1.73)	.74	—	—
Right sided only	0.18 (0.04–0.84)	.03	0.19 (0.03–1.09)	.06

Abbreviations: aOR, adjusted odds ratio; CI, confidence interval; IDU, injection drug use; OR, odds ratio.

The clinical outcomes shown in Table 4 demonstrate high rates of re-admission for endocarditis with 20% of those with IDU being re-admitted compared with 9% in the non-IDU patients. The majority of the re-admissions in the IDU group (13 of 21) were not persistent cases of endocarditis or treatment failure but were new infections. There was no significant difference in all-cause 1-year mortality in individuals with IDU-IE (16 [16%]) compared with those with non-IDU IE (37 [13%]). Of the 37 patients in the non-IDU IE group who were deceased at 1 year, only 3 had undergone surgery to treat their endocarditis. In contrast, the deaths in the IDU-IE group were equally distributed between the surgical and medical management arms, with 7 of 40 (18%) deceased at 1 year in the surgical group compared with 9 of 63 (14%) in the medical management group (*P* = .66).

**Table 4.** Clinical Outcomes of Patients With Infective Endocarditis

Clinical Outcomes	IDU-IE n = 103 (%)	Non-IDU IE n = 278 (%)	*P* Value
Re-admission for endocarditis	21 (20)	26 (9)	.004
Relapse or treatment failure	8 (8)	20 (7)	.85
Reinfection	13 (13)	6 (2)	<.0001
Death overall	25 (24)	70 (25)	.86
Death in hospital	6 (6)	25 (9)	.32
One-year mortality	16 (16)	37 (13)	.58

Abbreviations: IDU IE, injection drug use-related infective endocarditis.

Although 90 of the 103 IDU-IE patients met with social work, only 50 individuals were seen by either an addiction nurse specialist or a psychiatrist. Nine of the 96 patients (9%) who survived until discharge were started on medication to treat their opioid addiction, and all were alive at 1 year (Fisher exact test, *P* = .36). Among our discharged IDU-IE patients, 82 of 96 received their postdischarge antibiotic treatment at 1 of 2 state-run, long-term care facilities.

## Discussion

Infective endocarditis continues to be associated with significant morbidity and mortality [4, 12], and patients with a history of IDU-IE have been found to have an increased hazard of death or reoperation at 3–6 months postoperatively compared with those without an IDU history [6]. In this study, the unadjusted 1-year all-cause mortality rate was similar for patients with IDU-IE and non-IDU IE, despite IDU-IE patients being younger, having fewer comorbid conditions, and undergoing more frequent surgical interventions when indicated. The more aggressive surgical treatment of the IDU-IE population is notable given the rather similar distribution of EuroSCOREs in each cohort and reflects an optimal response to perceived surgical indications in this population. Of the 16 individuals with IDU-IE with an absolute surgical indication who did not proceed to surgery, 12 improved clinically and were discharged in stable condition with resolution of their heart failure or no further systemic emboli, whereas the other 4 were deemed too unstable for surgery and ultimately succumbed to their infection during their index hospitalization. The cause of postdischarge death in both the IDU-IE and non-IDU IE groups was largely unavailable, and although it is suspected that many with IDU-related endocarditis may die secondary to other complications of opioid use disorder (OUD), this is difficult to ascertain.

This study highlights the evolving trends in demographics, age, microbiology, and site of infection among patients with IDU-IE. In this cohort, 46% of IDU-IE patients were female, similar to a recent report of individuals hospitalized at community hospitals for IDU-IE between 2000 and 2013, wherein women accounted for 40.9% of cases of all IDU-IE and 53% of cases in the 15- to 34-year-old age group [13]. This trend demonstrating an increase in the proportion of females with IDU-IE likely is explained by national epidemiologic trends of substance use. When comparing 2011–2013 to 2002–2004, there has been a 100% increase in the average rates of woman who reported heroin use within the last year [14].

Among persons not injecting drugs, the frequency of endocarditis increases with age [4, 15, 16]. There has been an overall increase in the mean age of hospitalized patients with IE over the last 5 decades from 45.3 years in the 1980s to 57.2 years in the 2000s [16]. However, patients with IDU-IE are significantly younger than those with non-IDU IE. Our finding of a median age of 32 years is consistent with previous studies that report a mean age of 35–37 years among IDU-IE cohorts [17, 18].

The microbiology and site of infection with IE have changed over time. The incidence of *S aureus* has increased over the last half-century worldwide and is the most common cause of IE in developed countries [4, 19]. This is most evident among cases of IDU-IE [20]. In our study, there were as many cases of *S aureus* (65%) among patients with IDU-IE as there were cases of *S aureus* and *Streptococcal* spp combined (67%) in non-IDU IE. In both groups, those with and without IDU, *S aureus* isolates were more commonly methicillin susceptible than resistant, and, in particular, individuals with IDU-IE had MSSA infection approximately 2.5 times more frequently than MRSA. Infection with *S aureus* puts patients with IDU-IE at increased risk of severe sepsis, multiorgan dysfunction, major neurological events, and death [7, 9, 20]. In this report, patients with IDU-IE experienced significantly higher rates of vegetation >1 cm and extracardiac emboli compared with non-IDU patients.

The International Collaboration on Endocarditis-Prospective Cohort Study investigators found most cases of IE in a pooled international population involve vegetations on either the aortic (38%) or mitral valves (41%) compared with the tricuspid valve (12%) [4]. A relatively high rate of left-sided involvement in IDU-IE was also observed in our study and is further supported by recent studies evaluating long-term outcomes in IE with a focus on IDU [7, 20]. Infective endocarditis involving the left side of the heart is associated with increased mortality and need for cardiac surgery, particularly when the aortic valve is involved [18, 20].

A higher percentage of IDU-IE patients underwent cardiac surgery than did those in the non-IDU group (38% vs 28%). This trend was even more pronounced when there was an absolute surgical indication present (64% vs 43%). In the context of comparable EuroSCOREs, the surgery rates reflect a willingness to aggressively provide surgical treatment for the IDU-IE population. Age of 60 or less, large vegetation, paravalvular abscess, and increased EuroSCORE were associated with cardiac surgery within 90 days The 90-day window for cardiac surgical intervention was selected based on previous efforts to capture surgical interventions linked to the sentinel IE admission while limiting confounding surgical interventions for recurrent IE [6, 7]. As expected, the presence of exclusively right-sided disease was a predictor for medical management, which is consistent with current US guidelines [21].

In this study, both patient groups had high rates of re-admission for endocarditis; 9% of the non-IDU patients and 20% of those with IDU. Of note, however, the vast majority of the non-IDU re-admissions for endocarditis (20 of 26, 77%) were considered to have persistent or relapsed infection, meaning recurrence of same pathogen within 6 months of initial endocarditis. In contrast, among the IDU patients admitted for endocarditis, a majority of cases (13 of 21, 62%) had de novo infection (different pathogen or endocarditis greater than 6 months after initial event).

Our study has several limitations. It is a retrospective review performed at a single urban academic institution that may limit its generalizability to nonacademic, nonurban settings. Because this is a single-center study, patients may have received care at other institutions after sentinel admission at our facility, which could alter our surgical and IE re-admissions rates. If such occurred, however, it would only increase the rates or re-admission for endocarditis. Given that we have a much higher than national average percentage of patients whose endocarditis is related to IDU, our clinical care and willingness to perform surgical interventions may be different than that in other settings. Finally, we may have underestimated mortality, because the SSDI at the time of review only includes documented deaths through 2014. Therefore, we may have not accurately calculated mortality if our electronic medical record did not capture deaths occurring after 2014, particularly in patients who died out of the hospital, because most of our patients with IDU-IE were transfers from outside facilities. We recognize that the IDU-related deaths in particular may not be reported back to this medical center or seen in publicly available obituaries to a higher degree than the non-IDU IE group, thus underestimating the out-of-hospital mortality rate in the IDU-IE group.

The potential impact that treatment of OUD could have on the long-term outcomes of patients with IDU-IE represents a growing area of research and possibly one of the most significant opportunities to improve outcome. Englander et al [22] reported that a majority of individuals were interested in medications to treat addiction while hospitalized, and several publications have cited the inpatient setting as a place to intervene with addiction treatment [23–27]. The vast majority of our IDU-IE patients completed their antibiotic therapy at facilities that had the capacity to start treatment for OUD and link patients to continuing addiction care. This limits our ability to fully assess treatment interventions for addiction. Nevertheless, 9 of our discharged IDU-IE patients were offered medication to treat their OUD at our facility, and none of them had been re-admitted to our hospital for IDU-IE or had died at 1 year of follow up. Although our numbers are too small to be statistically meaningful, a recent report demonstrated decreased mortality in IDU-IE patients who received a referral for addiction treatment even though these referrals only occurred in 20% of the patients in this cohort [28]. This may represent the best evidence to date that optimal endocarditis therapy coupled with treatment of addiction can reduce mortality in patients with IDU-IE and potentially achieve long-term treatment outcomes that would be expected in young patients with few comorbidities being treated for this condition.

In spite of an increase in patients with IDU-IE, universal infectious disease subspecialty care, and a surgical service that has demonstrated a willingness to perform surgical procedures to treat IDU-IE patients with an absolute surgical indication, addiction treatment and harm reduction interventions are only beginning to be offered consistently in our setting. The challenge to provide wraparound services to those with substance use disorder, including prompt referral for addiction treatment, harm reduction interventions, and follow-up longitudinal care, will be critical to optimal management of OUD and complicating endocarditis or other infections. It is incumbent upon physicians treating patients with IDU-IE and other IDU-related infections to bridge this care if the annual number of opioid deaths, which are now projected to reach over 80 000 by 2025 [29], are to be reduced.

## Conclusions

In conclusion, IE remains a highly morbid condition, disproportionally so in patients with IDU-IE. Our data suggest that although patients with IDU-IE are much younger, have fewer comorbid conditions, and receive aggressive surgical management when indicated, they suffer mortality rates similar to a more at-risk non-IDU IE cohort. Further studies are warranted to investigate the more nuanced impact of IDU on the management of IE and whether additional interventions, particularly targeting underlying substance use disorder, can improve outcomes for patients with IDU-IE.
